# Liraglutide Alleviates Hepatic Steatosis by Activating the TFEB-Regulated Autophagy-Lysosomal Pathway

**DOI:** 10.3389/fcell.2020.602574

**Published:** 2020-11-27

**Authors:** Yunyun Fang, Linlin Ji, Chaoyu Zhu, Yuanyuan Xiao, Jingjing Zhang, Junxi Lu, Jun Yin, Li Wei

**Affiliations:** ^1^Shanghai Key Laboratory of Diabetes Mellitus, Department of Endocrinology and Metabolism, Shanghai Diabetes Institute, Shanghai Clinical Center for Diabetes, Shanghai Key Clinical Center for Metabolic Disease, Shanghai Jiao Tong University Affiliated Sixth People’s Hospital, Shanghai, China; ^2^National Demonstration Center for Experimental Fisheries Science Education, Shanghai Ocean University, Shanghai, China; ^3^Department of Endocrinology and Metabolism, Shanghai Eighth People’s Hospital, Shanghai, China

**Keywords:** non-alcoholic fatty liver disease, liraglutide, autophagy-lysosome, lysosomal biogenesis, TFEB

## Abstract

Liraglutide, a glucagon-like peptide-1 receptor agonist (GLP-1RA), has been demonstrated to alleviate non-alcoholic fatty liver disease (NAFLD). However, the underlying mechanism has not been fully elucidated. Increasing evidence suggests that autophagy is involved in the pathogenesis of hepatic steatosis. In this study, we examined whether liraglutide could alleviate hepatic steatosis through autophagy-dependent lipid degradation and investigated the underlying mechanisms. Herein, the effects of liraglutide on NAFLD were evaluated in a high-fat diet (HFD)-induced mouse model of NAFLD as well as in mouse primary and HepG2 hepatocytes exposed to palmitic acid (PA). The expression of the GLP-1 receptor (GLP-1R) was measured *in vivo* and *in vitro*. Oil red O staining was performed to detect lipid accumulation in hepatocytes. Electron microscopy was used to observe the morphology of autophagic vesicles and autolysosomes. Autophagic flux activity was measured by infecting HepG2 cells with mRFP-GFP-LC3 adenovirus. The roles of GLP-1R and transcription factor EB (TFEB) in autophagy-lysosomal activation were explored using small interfering RNA. Liraglutide treatment alleviated hepatic steatosis *in vivo* and *in vitro*. In models of hepatic steatosis, microtubule-associated protein 1B light chain-3-II (LC3-II) and SQSTM1/P62 levels were elevated in parallel to blockade of autophagic flux. Liraglutide treatment restored autophagic activity by improving lysosomal function. Furthermore, treatment with autophagy inhibitor chloroquine weakened liraglutide-induced autophagy activation and lipid degradation. TFEB has been identified as a key regulator of lysosome biogenesis and autophagy. The protein levels of nuclear TFEB and its downstream targets CTSB and LAMP1 were decreased in hepatocytes treated with PA, and these decreases were reversed by liraglutide treatment. Knockdown of TFEB expression compromised the effects of liraglutide on lysosome biogenesis and hepatic lipid accumulation. Mechanistically, GLP-1R expression was decreased in HFD mouse livers as well as PA-stimulated hepatocytes, and liraglutide treatment reversed the downregulation of GLP-1R expression *in vivo* and *in vitro*. Moreover, GLP-1R inhibition could mimic the effect of the TFEB downregulation-mediated decrease in lysosome biogenesis. Thus, our findings suggest that liraglutide attenuated hepatic steatosis via restoring autophagic flux, specifically the GLP-1R-TFEB-mediated autophagy-lysosomal pathway.

## Introduction

Non-alcoholic fatty liver disease (NAFLD), characterized by excessive triglyceride (TG) accumulation in the liver, is commonly associated with insulin resistance, obesity, diabetes, and cardiovascular disease (Arab et al., 2018). Currently, the prevalence of NAFLD is estimated to be approximately 10–35% in the general population, making it the most common liver disease worldwide (Vernon et al., 2011). Thus, there is an urgent need to develop new preventive and therapeutic strategies to alleviate NAFLD.

Liraglutide, a glucagon-like peptide-1 receptor agonist (GLP-1RA), exhibits beneficial effects on weight loss, cardiovascular function, and NAFLD in addition to its glucose-lowering effect, especially in patients with obesity (Drucker, 2018; Muller et al., 2019). Previous studies suggested that liraglutide could alleviate high-fat diet (HFD)−induced hepatic lipid accumulation in a weight loss-independent manner (Lyu et al., 2020). However, the mechanism underlying the effects of liraglutide on NAFLD remains unclear.

Autophagy is an important lysosome-mediated process for the degradation of cellular components, including damaged organelles and misfolded proteins, for the maintenance of intracellular energy homeostasis (Yang and Klionsky, 2010). Increasing evidence has demonstrated that autophagy is necessary for regulating lipid metabolism because lipid droplets are degraded through lipophagy (Singh et al., 2009; Cui et al., 2020). Disruption of autophagy might therefore contribute to the aggravation of hepatic lipid accumulation (Wu et al., 2020). Recent studies revealed that liraglutide could alleviate hepatic steatosis, reduce oxidative stress, and activate autophagy (Tong et al., 2016). However, the exact mechanisms underlying these effects have not been fully elucidated.

Lysosomes are organelles involved in the process of intracellular substrate degradation through autophagosome-lysosome fusion and play a critical role in regulating autophagic flux and lipid droplet clearance. Normally, lysosomal lipid degradation capacity depends on a sufficient number of lysosomes and the activation of acid hydrolase enzymes (Sinha et al., 2014; Pi et al., 2019). Transcription factor EB (TFEB), a key member of the microphthalmia family (MiT/TFE), has been identified as a key regulator of lysosome biogenesis and autophagy (Medina et al., 2011). Under normal conditions, TFEB is inactive and in the cytosol, whereas fasting and stress conditions induce its dephosphorylation-mediated activation and subsequent nuclear translocation. TFEB activation upon nuclear translocation may promote lysosomal biogenesis and substrate degradation (Zhang et al., 2018). Recently, TFEB has emerged as a key regulator of the lysosomal degradation of lipid droplets (Su et al., 2018). Moreover, deficient lysosomal clearance, resulting from reduced lysosome quantity and hydrolytic enzyme activity, was implicated in the development of NAFLD (Wang et al., 2019). These findings highlight a potential role of TFEB in the treatment of NAFLD via the promotion of autophagy-dependent lipid degradation. However, whether TFEB mediates the beneficial effect of liraglutide on hepatic autophagic flux and lipid degradation remains unclear.

In this study, we assessed the effects of liraglutide on hepatic steatosis and investigated the underlying molecular mechanisms. Our results revealed that autophagy activation mediated the effect of liraglutide in decreasing hepatic steatosis. Moreover, liraglutide activated autophagic flux and lipid degradation via the GLP-1R-TFEB-mediated autophagy-lysosomal pathway.

## Materials and Methods

### Animal Treatment

Eight-week-old C57BL/6J mice weighting 23–28 g were purchased from the Model Animal Research Center of Nanjing University. All animals were maintained in a specific pathogen-free barrier facility under a 12 h light/dark cycle. After a week of adaptation, the mice were randomly divided into two groups (*n* = 10). They were fed a normal chow diet (D12450J, Research Diets) or HFD (D12492, Research Diets) for 18 weeks. The mice were then intraperitoneally administered either liraglutide (400 μg/kg/day, Novo Nordisk, Denmark) or equivoluminal 0.9% saline for another 4 weeks. Body weight was measured weekly, and food intake was recorded every 3 days. All animal procedures were conducted according to the guidelines for Experimental Animal Research.

### Glucose Tolerance Test and Insulin Tolerance Test

The intraperitoneal glucose tolerance test (IPGTT) and insulin tolerance test (ITT) were performed to evaluate glucose tolerance and insulin sensitivity after 4 weeks liraglutide treatment. For IPGTT, the mice were intraperitoneally injected with glucose (2 g/kg body weight) after overnight fasting. For ITT, the mice were fasted for 6 h and injected with insulin (Humulin R, Eli Lilly and Company) at a concentration of 1 U/kg body weight. Blood glucose was monitored with a glucometer (Roche, Switzerland) at regular intervals (0, 15, 30, 60, 90, and 120 min).

### Biochemical and Lipid accumulation Measurements

The mice were sacrificed after an overnight fast. Serum triglyceride (TG), total cholesterol (TC), low-density lipoprotein cholesterol (LDL-C), alanine aminotransferase (ALT), and aspartic acid transaminase (AST) levels were examined using an automatic biochemistry analyzer (Rayto Technologies, China). The TG and TC of liver tissues and hepatocytes were measured using an Enzymatic Assay Kit (Applygen Technologies, China; Nanjing Jiancheng, China) according to the manufacturer’s protocol.

### Histopathological Analysis

Fresh liver specimens were fixed with 4% paraformaldehyde and embedded in paraffin. Paraffin sections were stained with H&E according to a standard procedure (Chang et al., 2010). Images were captured using a light microscope (Leica Microsystems, Germany).

### Oil Red O Staining and BODIPY 493/503 Staining

To assess hepatic steatosis, liver tissues were embedded in optimum cutting temperature and immediately frozen in liquid nitrogen. Frozen liver sections were stained with Oil Red O (O0625, Sigma-Aldrich) according to the manufacturer’s instructions. For *in vitro* studies, the hepatocytes were washed twice with phosphate-buffered saline and fixed in 4% paraformaldehyde for 30 min. Fixed cells were stained with Oil Red O solution for 0.5–1 h and gently washed with 60% isopropanol. Three images per sample were captured using a light microscope (Leica Microsystems, Germany). Lipid droplets were also detected by labeling with the BODIPY 493/503 (D3922, Invitrogen, United States) lipid dye. The images were obtained using a confocal microscope (Carl Zeiss, Germany).

### Electron Microscopy

Autophagic vacuoles and autolysosomes were observed using an electron microscope (Olympus, Japan). Fresh liver tissue pieces were fixed with 2.5% glutaraldehyde, dehydrated in a graded ethanol series, and embedded in Epon 812 (Electron Microscopy Sciences, United States).

### Cell Culture and Treatments

Human hepatoma HepG2 cells were obtained from the Type Culture Collection of the Chinese Academy of Sciences (Shanghai, China). Murine primary hepatocytes were isolated from male C57BL/6J mice (8 weeks old) using the collagenase IV perfusion technique as previously described (Seglen, 1976; Klaunig et al., 1981). All cells were cultured in DMEM/HIGH GLUCOSE medium containing 10% fetal bovine serum (FBS) and 1% penicillin-streptomycin (Gibco, United States) in a 5% CO_2_ incubator at 37°C. To establish a lipid-loaded cell model mimicking NAFLD *in vitro*, palmitic acid (PA, P9767, Sigma-Aldrich) was added to the culture medium at a final concentration of 0.2–0.6 μM. For *in vitro* experiments, cells were incubated in serum-free medium overnight and were then treated with or without liraglutide (S8256, Selleck Chemicals; 100–500 nM) in serum-free media containing PA or vehicle for 24 h. To examine the effect of liraglutide on hepatic autophagic flux, chloroquine (CQ, C6628, Sigma Aldrich; 50 μM), a classical autophagy inhibitor that blocks the fusion of autophagosomes with lysosomes, was added to the medium for 4–6 h as a negative control. To investigate the TFEB-mediated effects of liraglutide on lysosome biogenesis and autophagy, HepG2 cells were transfected with small interfering RNA (siRNA) targeted against TFEB (Genepharma, China) for 48 h using Lipofectamine 2000 (Thermo Fisher Scientific, United States). The siRNA sequences used are as follows: sense 5′-GACGAAGGUUCAACAUCAATT-3′ and antisense 5′-UUGAUGUUGAACCUUCGUCTT-3′. To further investigate the mechanism of TFEB nuclear translocation, HepG2 cells were transfected with GLP-1R plasmid (Hanbio Biotechnology, China) and small interfering RNA (siRNA) targeted against GLP-1R (Genepharma, China). The siRNA sequences used were as follows: sense 5′-GGACCAGGAACUCCAACAUTT-3′ and antisense 5′-AUGUUGGAGUUCCUGGUCCTT-3′.

### Western Blot and Immunofluorescence Analysis

Total protein from liver tissue and hepatocytes was extracted with RIPA Lysis Buffer (P0013B, Beyotime, China) at 4°C. Nuclear and cytoplasmic fractions were separated for analysis of TFEB translocation using a commercial kit (78835, Thermo Fisher Scientific, United States) according to the manufacturer’s instructions. Protein samples (20–30 μg) were separated using SDS-PAGE (Bio-Rad Laboratories, United States) and transferred to PVDF membranes (Millipore Corporation, United States). The membranes were blocked with 5% skimmed milk and incubated with primary antibody for 12 h followed by incubation with horseradish peroxidase-conjugated secondary antibodies. Primary antibodies against LC3A/B (1:1,000, 12741), Beclin1 (1:1,000, 3495), Atg5 (1:1,000, 12994), Atg7 (1:1,000, 8558), GAPDH (1:1,000, 2118s), cathepsin B (1:1,000, 31718), p62 (1:1,000, 5114s), and β-actin (1:1,000, 4970s) were obtained from Cell Signaling Technology (United States). Antibodies against lysosome-associated membrane protein 1 (LAMP1) (1:1,000, sc-20011) and SIRT1 (1:1,000, sc-15404) were from Santa Cruz Biotechnology, and the anti-TFEB (1:2,000, A303-673A) antibody was from Bethyl Laboratories (United States). The antibody against GLP-1R (1:1,000, ab39072) was from Abcam (United Kingdom). Membrane signals were detected using an enhanced chemiluminescence method (Thermo Fisher Scientific, United States). The band intensities were quantitated using ImageJ software. For immunofluorescence, cells were fixed with 4% paraformaldehyde for 15 min at 37°C. After fixation, the cells were permeabilized with 0.25% Triton X-100 for 15 min, blocked with 2% BSA for 30 min, and then incubated with an anti-TFEB antibody (1: 100, MAB9170-100, R&D Systems, United States) overnight at 4°C. The next day, cells were stained with a secondary antibody for 1 h and DAPI for 5 min. Immunofluorescence was visualized using a fluorescent microscope (Leica Microsystems, Germany).

### Analysis of Autophagic Flux

For autophagic flux measurement, human hepatoma HepG2 cells were transfected with adenovirus harboring mRFP-GFP-LC3 (Hanbio Biotechnology, China). The cells were then treated with 0.4 μM PA, liraglutide, or CQ for 24 h. After treatment, yellow and red puncta were detected using a confocal microscope (Carl Zeiss, Germany).

### Statistical Analysis

The data are expressed as the mean ± standard error of the mean (SEM). Unpaired two-tailed *t*-tests were used for intergroup comparisons. One-way analysis of variance with *post hoc* Bonferroni tests was performed for comparisons between multiple groups.

## Results

### Liraglutide Improves Systemic Glucose and Lipid Homeostasis in HFD-Fed Mice

**FIGURE 1:**
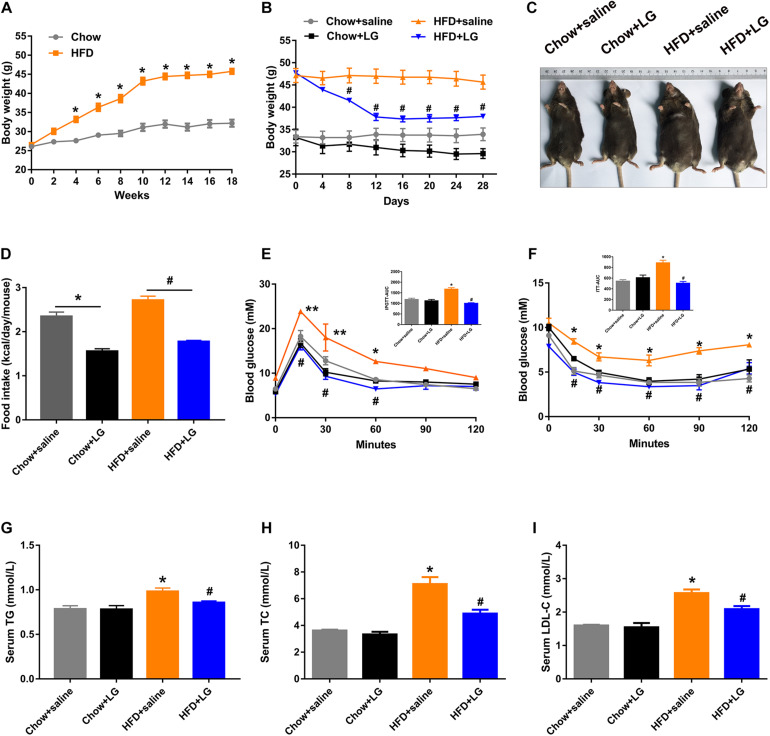
Liraglutide improves systemic glucose and lipid metabolism in HFD mice. **(A)** Body weight before intervention. **(B)** Body weight during liraglutide treatment. **(C)** Representative photos of chow diet- and HFD-fed mice treated with liraglutide (400 μg/kg) or saline daily for 4 weeks. **(D)** Food intake (kcal/day/mouse). **(E,F)** IPGTT and ITT results were obtained after liraglutide treatment. AUC: the area under the curve. **(G–I)** Serum triglycerides (TG), cholesterol (TC), and LDL-C levels. The data are expressed as mean ± SEM; *n* = 3–5. *P < 0.01, **P < 0.05 vs. Chow + saline group; ^#^*P* < 0.01 vs. HFD + saline group.

To explore the beneficial effect of liraglutide on hepatic lipid accumulation, C57BL/6 mice were fed a HFD or chow diet for 18 weeks and were then administered liraglutide (400 μg/kg/day) or saline for another 4 weeks. From the 4th week of intervention, HFD-fed mice exhibited a higher body weight than control mice, which was the case until the end of the experiment (Figure 1A). Liraglutide treatment significantly decreased body weight in HFD-fed mice (Figure 1B[[AQ11]]). Total food intake was decreased in both liraglutide-treated controls and HFD-fed mice (Figure 1D). Further, although liraglutide treatment induced a transient reduction of food intake during the first 2 weeks, no significant differences were found between the HFD groups treated with or without liraglutide during 3–4 weeks (Supplementary Figure S1). In addition, GTT and ITT indicated that liraglutide alleviated glucose intolerance and insulin resistance in HFD-fed mice (Figures 1E,F). However, no significant differences were observed in GTT results between these groups after blood glucose was adjusted as a% of initial glucose (Supplementary Figures S2A,B). Regarding systemic lipid metabolism, as shown in Figures 1G–I, the serum levels of TG, TC, and LDL-C in HFD-fed mice were decreased by liraglutide treatment. Taken together, these results suggest that liraglutide reduced body weight and restored systemic glucose and lipid homeostasis in diet-induced obese mice.

### Liraglutide Mitigates HFD-Induced Hepatic Steatosis and Liver Injury

**FIGURE 2:**
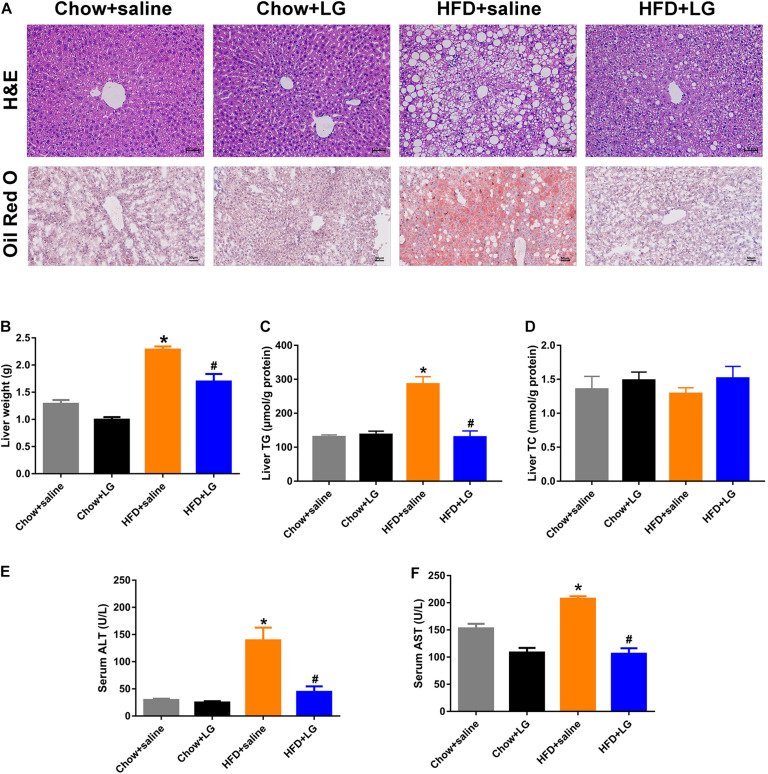
Liraglutide attenuates hepatic steatosis and liver injury in HFD mice. **(A)** H&E and Oil red O staining of liver tissue (scale bars = 50 μm). **(B)** Liver weight (g). **(C,D)** Liver TG and TC levels. **(E,F)** Serum ALT and AST levels. The data are expressed as mean ± SEM; *n* = 3–5. *P < 0.01 vs. Chow + saline group; ^#^*P* < 0.01 vs. HFD + saline group.

Since liraglutide improved systemic lipid homeostasis, we further investigated its effects on HFD-induced hepatic steatosis. H&E and Oil red O staining revealed that liraglutide treatment visibly attenuated HFD-induced hepatic lipid accumulation, with a reduced number of intracellular lipid droplets and ballooning hepatocytes (Figure 2A). Consistent with these changes, liver weight and hepatic TG content in HFD mice were significantly higher than those in chow diet-fed mice. Liraglutide also decreased liver weight and hepatic TG accumulation in HFD-fed mice. No significant differences were observed in TC content between these groups (Figures 2B–D). Biochemical analysis revealed that elevated serum ALT and AST levels in HFD group mice were clearly decreased by liraglutide (Figures 2E,F). Collectively, these results indicated that liraglutide exerted its hepatoprotective effects by alleviating hepatic steatosis and liver injury.

### Liraglutide Attenuates PA-Induced Lipid Accumulation *in vitro*

**FIGURE 3:**
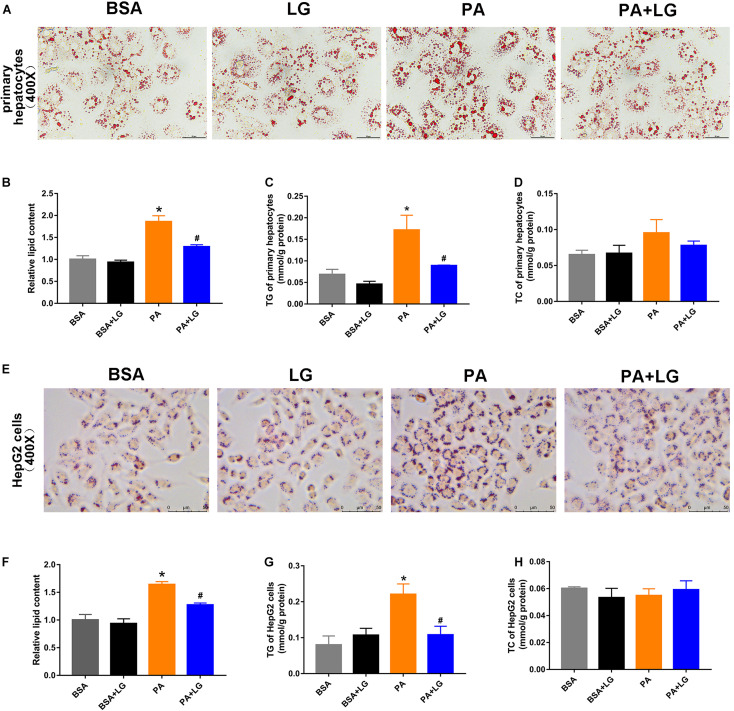
Liraglutide attenuates PA-induced lipid accumulation in primary mouse hepatocytes and HepG2 cells. **(A,E)** Primary mouse hepatocytes and HepG2 cells were co-incubated with PA and liraglutide for 24 h. Lipid accumulation was assessed by Oil red O staining (scale bars = 50 μm). **(B,F)** Relative lipid content. **(C,G)** Hepatocyte TG content. **(D,H)** Hepatocyte TC content. The data are expressed as mean ± SEM; *n* = 3. *P < 0.05 vs. BSA group; ^#^*P* < 0.05 vs. PA group.

To confirm whether liraglutide could alleviate hepatic lipid accumulation *in vitro*, primary mouse hepatocytes and HepG2 cells were employed. As shown in Figures 3A,E, lipid accumulation was significantly increased in both PA-stimulated primary mouse hepatocytes and HepG2 cells, an effect which was suppressed by co-incubation with liraglutide. Liraglutide treatment also significantly decreased intracellular TG content in these cell models (Figures 3C,G). However, no significant differences were observed in TC content (Figures 3D,H). Our results revealed that liraglutide directly reduced hepatic steatosis *in vitro*.

### Liraglutide Induces Autophagy in HFD Mice

**FIGURE 4:**
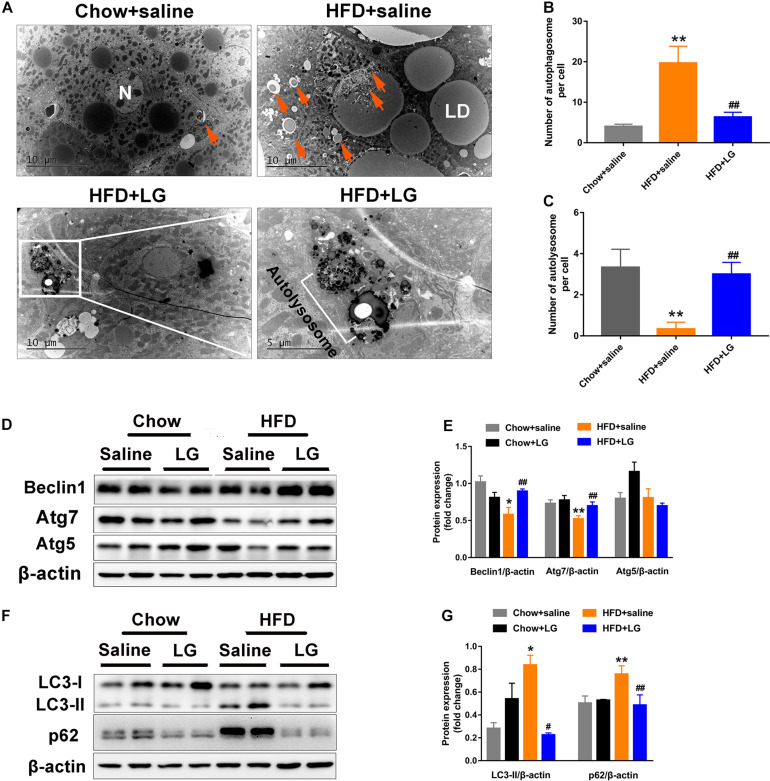
Liraglutide activates autophagy in HFD-fed mice. **(A)** Representative electron microscopy images of autophagic vacuoles in the liver. Orange arrows indicate autophagic vacuoles (partly containing lipid droplets). Boxed area indicates autolysosome. High magnification of boxed areas in the liraglutide treatment group is presented on the right (scale bars = 10, 5 μm). N, nucleus; LD, lipid droplet; arrows, autophagosome; white box, autolysosome. **(B)** Quantitative analysis of number of autophagosomes per cell. **(C)** Quantitative analysis of number of autolysosomes per cell. **(D,E)** Expression of autophagy-associated proteins Atg5, Atg7, and Beclin1 in livers of HFD-fed mice analyzed by western blot. **(F,G)** Expression of autophagy-associated proteins LC3-II and autophagic selective substrate p62 in livers of HFD-fed mice analyzed by western blot. The data are expressed as mean ± SEM; *n* = 3. *P < 0.01, **P < 0.05 vs. Chow + saline group; ^#^*P* < 0.01 ^##^*P* < 0.05 vs. HFD + saline group.

To explore whether liraglutide alleviated hepatic steatosis in HFD mice by activating autophagy, EM was performed. We observed large numbers of lipid droplets and autophagosome-like vesicles in the livers of HFD mice. These vesicles might derive from the sequestration of small lipid droplets or a portion of large lipid droplets, suggestive of lipid-laden autophagosomes. In contrast, virtually no lipid droplets were observed in liver sections from liraglutide-treated HFD mice, as intracellular lipids were engulfed and degraded within the autolysosome compartment (Figures 4A–C). Western blot revealed that the levels of autophagy indicators Atg7 and Beclin1 were lower in HFD mice, while those of LC3-II and autophagic selective substrate SQSTM1/p62 were significantly higher, indicating a blockade of autophagy-lysosomal degradation. Liraglutide treatment restored HFD-inhibited autophagy by increasing the protein expression of Atg7 and Beclin1 (Figures 4D,E) and decreasing the expression of LC3-II and p62 (Figures 4F,G). These findings suggest that hepatic steatosis impaired autophagy degradation, while liraglutide alleviated lipid accumulation by promoting autophagy-lysosomal−dependent lipid degradation.

### Liraglutide Attenuates PA-Induced Lipid Accumulation by Enhancing Autophagic Flux

**FIGURE 5:**
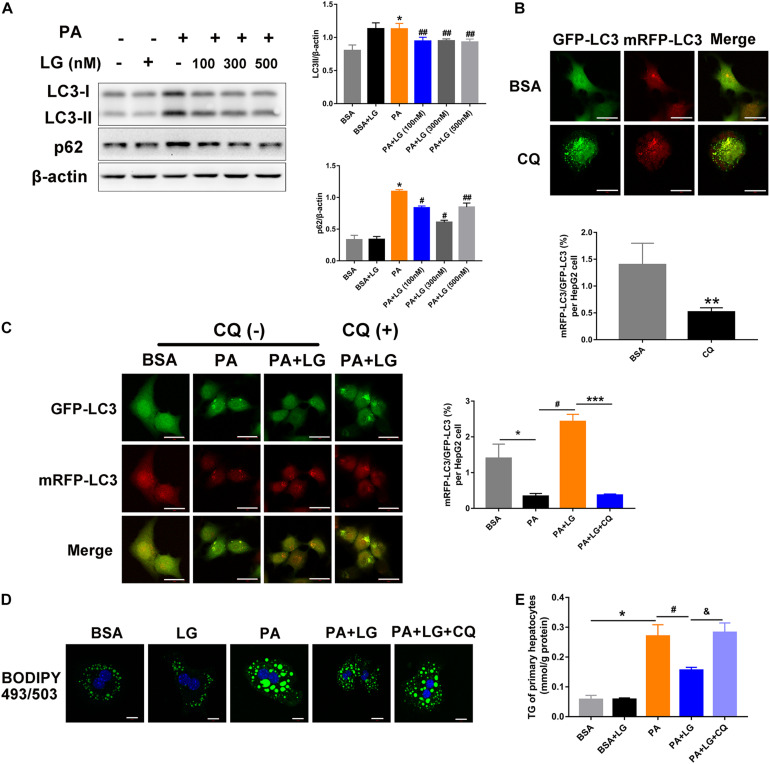
Liraglutide attenuates lipid accumulation by enhancing autophagic flux *in vitro*. **(A)**. Primary mouse hepatocytes were co-incubated with PA and different concentrations of liraglutide for 24 h. Expression of LC3-II and p62 was analyzed by western blot. **(B)**. Evaluation of autophagic flux in Ad-mRFP-GFP-LC3-infected cells with or without CQ treatment. **(C)**. After Ad-mRFP-GFP-LC3 infection, HepG2 cells were pretreated with PA and liraglutide for 20 h, followed by 4 h of co-treatment with 50 μM of CQ. The ratio of mRFP to GFP per cell (*n* = 10) was calculated (scale bars = 20 μm). **(D)** Lipid droplets were detected by labeling with lipid dye BODIPY 493/503 (green) in primary mouse hepatocytes treated with or without liraglutide and CQ. Nuclei were stained with DAPI (blue) (scale bars = 10 μm). **(E)** Intracellular TG content was measured. The data are expressed as mean ± SEM. *P < 0.01, **P < 0.05 vs. BSA group; ***P < 0.01 vs. PA + LG group; ^#^*P* < 0.01,^ ##^*P* < 0.05 vs. PA group; ^&^*P* < 0.01 vs. PA + LG group.

To further explore the mechanism underlying the liraglutide-mediated alleviation of hepatic steatosis via autophagic flux activation, primary mouse hepatocytes were isolated and incubated with free fatty acids. An increase in LC3-II and p62 levels was observed in hepatocytes after PA treatment, whereas oleic acid (OA) had no effect on the expression of these proteins (Supplementary Figure S3A). Furthermore, the expression of LC3-II and p62 increased in PA-stimulated primary mouse hepatocytes in a dose-dependent manner (Supplementary Figure S1B). However, the increase was reversed by liraglutide, indicating that liraglutide alleviated the PA−triggered dysfunction of autolysosomal degradation (Figure 5A). Moreover, HepG2 cells were infected with mRFP-GFP-LC3 adenovirus to confirm whether liraglutide could promote autophagic flux (Figures 5B,C). When an Ad-mRFP-GFP-LC3 construct is used, GFP fluorescence only indicates autophagosomes, since it can be easily quenched in the acidic pH of autolysosomes. However, mRFP detects both autophagosomes and autolysosomes, since it is more stable under acidic conditions. Using this approach, we observed that the ratio of mRFP to GFP signals was significantly decreased in PA-stimulated HepG2 cells, suggesting the accumulation of non-acidic autophagosomes. In liraglutide−treated cells, more mRFP signals were observed, indicating that autophagic flux was enhanced without impeding autophagosome-lysosome fusion/or autolysosome function. In addition, blocking fusion by CQ weakened the liraglutide-increased ratio of mRFP to GFP signals.

We then investigated whether liraglutide-induced autophagy was involved in its beneficial effect on lipid accumulation. As shown in Figures 5D,E, liraglutide treatment decreased BODIPY 493/503 fluorescence intensity and intracellular TG content in primary hepatocytes. However, these improvements were significantly diminished with CQ. Together, our results indicate that liraglutide alleviated hepatic steatosis partially by promoting the autophagy-lysosomal pathway.

### Liraglutide Stimulates Lysosome Biogenesis by Inducing TFEB Translocation

**FIGURE 6:**
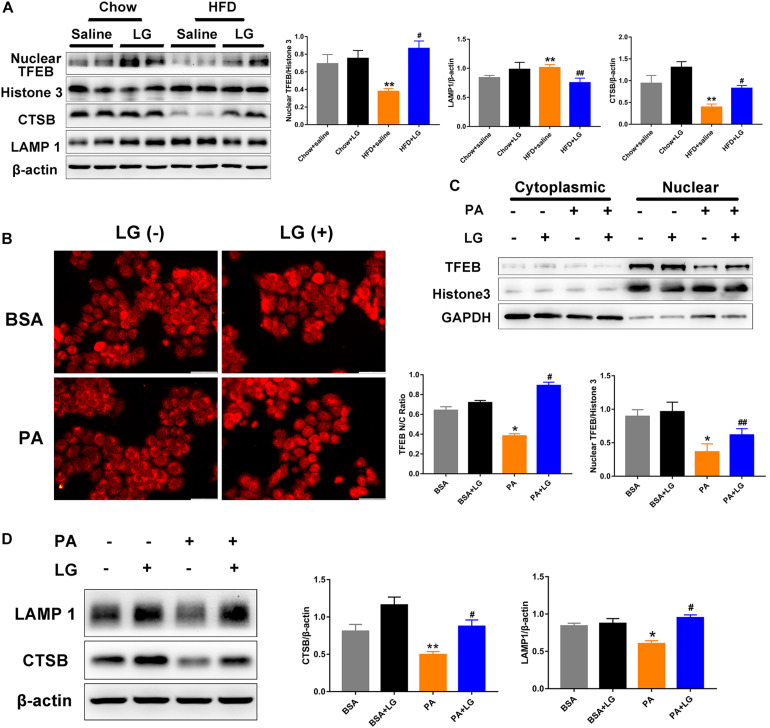
Liraglutide stimulates lysosome biogenesis by inducing TFEB nuclear translocation. **(A)** Expression of nuclear TFEB, CTSB, and LAMP1 in the livers of HFD-fed mice treated with or without liraglutide. Relative expression levels were normalized to β-actin and Histone 3 levels. **(B)** HepG2 cells were co-incubated with or without PA and liraglutide for 24 h. After fixation, immunofluorescence staining was performed for TFEB localization (scale bars = 50 μm). **(C)** Western blot detection of cytoplasm and nuclear TFEB expression in HepG2 cells treated with or without liraglutide. Relative expression levels were normalized to GAPDH and Histone 3 levels, respectively. **(D)** Western blot detection of CTSB and LAMP1 expression in HepG2 cells. The data are expressed as mean ± SEM; *n* = 3. *P < 0.01, **P < 0.05 vs. BSA group; ^#^*P* < 0.01, ^##^*P* < 0.05 vs. PA group.

Given that TFEB plays a critical role in the regulation of autophagy and lysosome biogenesis, we evaluated whether liraglutide alleviated steatosis by promoting TFEB-dependent lysosome biogenesis *in vivo* and *in vitro*. Upon its dephosphorylation and nuclear translocation, TFEB activates lysosome-associated gene transcription. Thus, we used subcellular fractionation to confirm its localization. Western blot revealed that nuclear TFEB and its downstream target CTSB were remarkably decreased in the livers of HFD-fed mice, and these decreases were reversed by liraglutide treatment. In contrast, LAMP1 expression was increased, possibly due to a compensatory response for autophagy inhibition (Figure 6A). Consistently, we observed that liraglutide promoted TFEB nuclear translocation and enhanced CTSB and LAMP1 expression in PA−stimulated HepG2 cells (Figures 6C,D). In addition, immunofluorescent results revealed that liraglutide increased the ratio of nuclear to cytosolic TFEB in HepG2 cells (Figure 6B). Overall, these findings suggest that liraglutide stimulated lysosome biogenesis by inducing TFEB nuclear translocation.

### Liraglutide Attenuates PA-Induced Lipid Accumulation by Activating TFEB-Mediated Lysosome Biogenesis

**FIGURE 7:**
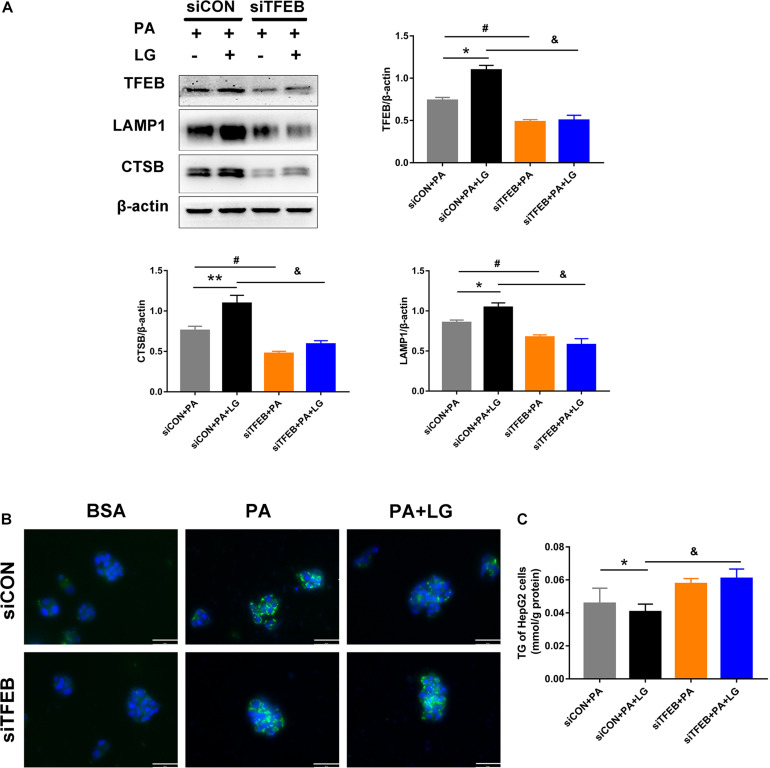
TFEB knockdown weakens the effects of liraglutide on lysosome biogenesis and lipid accumulation. **(A)** Western blot detection of TFEB and its downstream targets CTSB and LAMP1 in HepG2 cells expressing TFEB-siRNA or control-siRNA. Relative expression levels were normalized to β-actin levels. **(B)** Lipid droplets in HepG2 cells treated with TFEB-siRNA or control-siRNA were detected via labeling with the lipid dye BODIPY 493/503 (green). Nuclei were stained with DAPI (scale bars = 50 μm). **(C)** The TG content of HepG2 cells. The data are expressed as the mean ± SEM; *n* = 3. *P < 0.01, **P < 0.05 and ^#^*P* < 0.01 vs. siCON + PA group; ^&^*P* < 0.01 vs. siCON + PA + LG group.

To explore whether TFEB was responsible for the beneficial effects of liraglutide on lysosome biogenesis and lipid accumulation in hepatocytes, a TFEB siRNA (siTFEB) was used to silence TFEB in HepG2 cells. Results showed that TFEB downregulation decreased the expression of CTSB and LAMP1 in liraglutide-treated HepG2 cells (Figure 7A). In addition, decreased BODIPY 493/503 fluorescence intensity and TG content with liraglutide treatment in HepG2 cells were not observed under TFEB knockdown (Figures 7B,C). Our results demonstrate that TFEB plays a critical role in lysosome biogenesis and mediates the beneficial effect of liraglutide on PA-induced intracellular lipid accumulation.

### Liraglutide Activates TFEB and Its Downstream Targets Through GLP-1R

**FIGURE 8:**
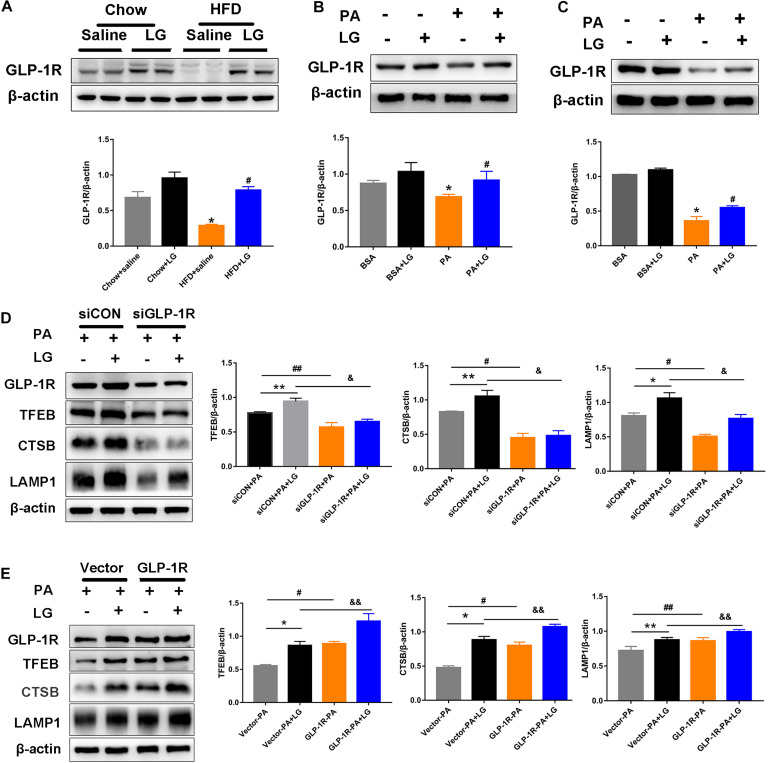
Liraglutide activates TFEB and its downstream targets through GLP-1R. **(A)** Western blot analysis of GLP-1R protein levels in the liver of HFD-fed mice treated with or without liraglutide. Relative expression levels were normalized to β-actin levels. **(B,C)** Western blot detection of GLP-1R in primary mouse hepatocytes and HepG2 cells treated with or without liraglutide. **(D)** Western blot detection of GLP-1R, TFEB, and its downstream targets CTSB and LAMP1 in HepG2 cells transfected with TFEB-siRNA or control-siRNA. **(E)** Western blot detection of GLP-1R, TFEB, and its downstream targets CTSB and LAMP1 in HepG2 cells with or without GLP-1R overexpression. The data are expressed as the mean ± SEM; *n* = 3. *P < 0.01, **P < 0.05 vs. Chow + saline group or BSA group or siCON + PA group or Vector-PA group; ^#^*P* < 0.01,^ ##^*P* < 0.05 vs. HFD + saline group or PA group or siCON + PA group or Vector-PA group; ^&^*P* < 0.01, ^&&^*P* < 0.05 vs. siCON + PA + LG group or Vector-PA + LG group.

To evaluate whether GLP-1R was involved in the action of liraglutide, the GLP-1R protein levels in livers and hepatocytes were detected by western blotting. GLP1-R expression was significantly decreased in HFD mouse livers and PA−stimulated hepatocytes. Further, liraglutide treatment reversed the downregulation of GLP-1R expression *in vivo* and *in vitro* (Figures 8A–C). To further investigate whether GLP-1R mediated the effect of liraglutide on TFEB-induced lysosome biogenesis, a GLP-1R siRNA (siGLP-1R) was used to silence GLP-1R in HepG2 cells. Results revealed that the downregulation of GLP-1R decreased the expression of TFEB and its downstream targets CTSB and LAMP1 in liraglutide-treated HepG2 cells (Figure 8D). Simultaneously, a GLP-1R plasmid was used to overexpress GLP-1R in HepG2 cells. GLP-1R overexpression enhanced the TFEB-mediated increase of CTSB and LAMP1 expression in PA-treated HepG2 cells (Figure 8E). Taken together, our results suggest that liraglutide upregulates TFEB and its downstream target proteins through GLP-1R.

## Discussion

**FIGURE 9:**
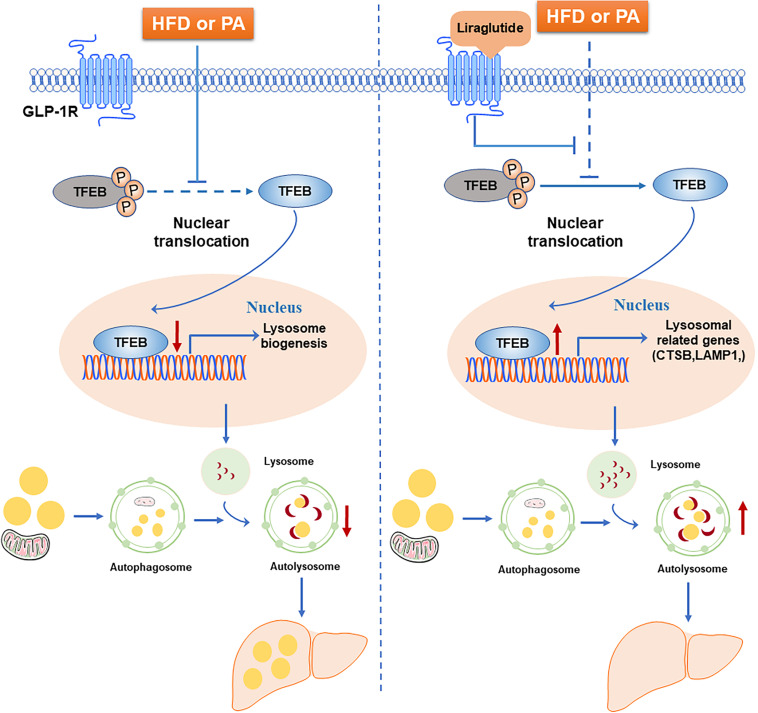
Schematic diagram of the mechanism of liraglutide-mediated alleviation of hepatic steatosis. HFD or PA exposure inhibits TFEB dephosphorylation and nuclear translocation. TFEB regulates lysosomal biogenesis and function. The blocking of TFEB nuclear translocation leads to autophagic flux impairment and subsequently aggravates hepatic steatosis. Liraglutide, a GLP-1R agonist, alleviates hepatic steatosis through enhancing autophagic flux. Mechanistically, liraglutide activates TFEB and its downstream targets through activation of GLP-1R.

In this study, we examined the beneficial effect of liraglutide on hepatic steatosis in HFD-fed mice and hepatocytes. Our results demonstrated that liraglutide alleviated hepatic lipid accumulation through activation of the autophagy-lysosomal pathway. Further, we found that, through GLP-1R activation, liraglutide promoted the nuclear translocation of TFEB as well as the expression of its targets CTSB and LAMP1, associated with lysosomal biogenesis and the autophagosome-lysosomal pathway. The increased nuclear translocation of TFEB reduced lipid accumulation in hepatocytes (Figure 9).

As novel potential therapeutic agents for NAFLD, GLP-1RAs were reported to reduce hepatic lipid accumulation (Wu et al., 2019). Further, clinical trial results revealed that liraglutide decreased liver fat content and body weight in patients with type 2 diabetes and NAFLD (Petit et al., 2017). GLP-1RAs also attenuated lipid accumulation in the livers of various animal models (Zheng et al., 2017; Kalavalapalli et al., 2019). These findings were consistent with our results, which indicated that liraglutide significantly decreased liver lipid content as well as plasma TG, ALT, and AST in HFD-fed mice. Another report described a number of effects for GLP-1RAs, including increased postprandial insulin secretion, decreased appetite, and enhanced weight loss (Armstrong et al., 2016). Previous studies suggested that the beneficial effect of GLP-1RAs on weight loss is mainly attributed to the inhibition of food intake, especially for short-term interventions (Kanoski et al., 2011; Wei et al., 2015). A recent research on exendin-4 treatment in mice revealed a decrease in food intake during the first 2 weeks, which disappeared from 2 to 8 weeks (Xu et al., 2016). Consistently, in our study, we observed that the transient reduction in food intake vanished in the last 2 weeks of liraglutide treatment. Thus, we speculated that the weight loss resulting from GLP-1RAs was not entirely dependent on the reduced food intake during long-term administration. Moreover, we investigated the expression of GLP-1R. Interestingly, liraglutide treatment reversed the down-regulation of GLP-1R expression in both *in vivo* and *in vitro* NAFLD models. Therefore, the systemic metabolic effects of GLP-1RAs might be attributed to a combination of the above-described mechanisms (Zhou et al., 2019; Mantovani et al., 2020).

Autophagy is important for the maintenance of cellular metabolism and energy homeostasis. Accumulating evidence has indicated that defective autophagy contributes to the aggravation of hepatic steatosis (Miyagawa et al., 2016; Chu et al., 2019). Normally, autophagy is supposed to break down lipid droplets through the lipophagy degradation pathway (Martinez-Lopez and Singh, 2015). A previous study found that GLP-1RA-induced autophagy could remove excess lipid droplets and thus alleviate lipotoxicity (Yu et al., 2019). Moreover, exendin-4 protected hepatocytes and reduced steatosis via autophagy, in turn reducing endoplasmic reticulum stress-related hepatocyte apoptosis (Sharma et al., 2011). However, the mechanism underlying this upregulation of autophagy is unclear. A previous study in mice demonstrated that GLP-1RAs alleviated lipid accumulation through activation of AMPK/mTOR-dependent autophagy, a classic autophagy pathway (He et al., 2016). In the current work, EM examination revealed a large number of lipid droplets surrounded or partly engulfed by autophagosome-like vesicles in the livers of HFD mice. Further, instead of these lipid droplets, lipid-laden autolysosomes were observed following liraglutide treatment. Consistently, the levels of autophagosome formation marker LC3−II and autophagic substrate p62 decreased after treatment with liraglutide. Thus, we inferred that liraglutide restored HFD-inhibited autophagy, perhaps in an autophagy-lysosomal pathway-dependent manner.

Autophagy is a dynamic process. As the terminal stage of autophagic flux, lysosomes fuse with autophagosomes, followed by lysosomal degradation and cellular lipid clearance (Chao et al., 2018a). Defective lysosomal biogenesis and clearance impair autophagic flux, leading to intracellular lipid accumulation during the development of NAFLD (Chao et al., 2018b). Previous studies found that HFD disrupted lysosomal biogenesis in hepatocytes, which was associated with lipid degradation (Pi et al., 2019). We employed lysosomal inhibitor CQ to further investigate autophagy-lysosomal pathway involvement and found that liraglutide stimulated autophagic flux, as demonstrated by a reduction in autophagosome accumulation indicated by mRFP-GFP-LC3. In addition, the liraglutide-mediated attenuation of lipid accumulation in PA-stimulated HepG2 cells was significantly compromised following CQ treatment. Importantly, this is the first work to demonstrate that liraglutide activates the autophagy-lysosomal pathway to alleviate hepatic lipid accumulation.

MiT−TFE subfamily members, including MITF, TFE3, and TFEB, positively regulate lysosomal biogenesis. Among these transcriptional factors, TFEB enhances lipid metabolism by regulating genes that encode lysosome biogenesis- and lipolysis-related factors (Napolitano and Ballabio, 2016). Under nutrient-depleted conditions, TFEB was activated by an autoregulatory feedback loop and subsequently regulated cellular lipid catabolism (Settembre et al., 2013). Recent studies have revealed that TFEB nuclear translocation was promoted by pharmacologically activating the AMPK pathway and eventually alleviated hepatic steatosis in HFD-fed mice (Chen et al., 2019). In addition, TFEB regulated multiple steps of lipid metabolism, such as lipid recruitment and degradation, as well as fatty acid oxidation (Li et al., 2016; Evans et al., 2019). However, the exact influence of liraglutide on TFEB-mediated lysosomal biogenesis had not been studied previously. Our study firstly revealed that liraglutide activated autophagic flux by inducing TFEB nuclear translocation and increasing the expression of its downstream targets CTSB and LAMP1. After silencing TFEB, the liraglutide-mediated promotion of lysosome biogenesis and alleviation of hepatic steatosis were attenuated. Moreover, we found that silencing of GLP-1R could mimic the effects TFEB downregulation in decreasing lysosome biogenesis, whereas its overexpression increased TFEB activation and the expression of its downstream targets CTSB and LAMP1, indicating that GLP-1R mediated the effect of liraglutide in promoting lysosome biogenesis. Therefore, we speculated that liraglutide activates the autophagy-lysosomal pathway to reduce hepatic lipid accumulation through GLP-1R-mediated TFEB activation.

In response to nutrients, GLP-1RAs were also found to alleviate liver steatosis through the upregulation of SIRT1 expression, activating the AMPK pathway and inhibiting SREBP-1c expression (Xu et al., 2014). Moreover, data from recent studies demonstrated that AMPK/SIRT1 pathway activation could upregulate the expression of various autophagy-related genes (Song et al., 2015; Huang et al., 2017). For instance, SIRT1 deacetylation could induce autophagy in an autophagy protein 5-dependent manner (Sun et al., 2018). In the current work, we found that liraglutide decreased SIRT1 expression in PA-treated primary mouse hepatocytes (Supplementary Figure S4C). However, the opposite results were observed in mice, which requires further investigation.

## Conclusion

In conclusion, our study reveals that liraglutide attenuates hepatic steatosis via activation of autophagic flux, especially the GLP-1R-TFEB-mediated autophagy-lysosomal pathway. These findings provide a novel mechanism supporting the potential of GLP-1RAs as promising therapeutic agents for NAFLD.

## Data Availability Statement

The original contributions presented in the study are included in the article/Supplementary Material, further inquiries can be directed to the corresponding authors.

## Ethics Statement

The animal study was reviewed and approved by Animal Ethical and Welfare Committee of Shanghai Sixth People’s Hospital Affiliated to Shanghai Jiao Tong University School.

## Author Contributions

YF and LW designed the study. YF and LJ carried out the experimental work. CZ and YX assisted in performing research. JZ and JL collected and analyzed the data. YF interpreted the results and wrote the manuscript. LW and JY contributed to manuscript read and revision. All authors have read and approved the submitted version.

## Conflict of Interest

The authors declare that the research was conducted in the absence of any commercial or financial relationships that could be construed as a potential conflict of interest.

## Abbreviations

GLP-1RA: glucagon-like peptide-1 receptor agonist
NAFLD: non-alcoholic fatty liver disease
GLP-1R: glucagon-like peptide-1 receptor
PA: palmitic acid
TFEB: transcription factor EB
siRNA: small interfering RNA
LC3-II: microtubule-associated protein 1B light chain-3-II
HFD: high-fat diet
IGPTT: intraperitoneal glucose tolerance test
ITT: insulin tolerance test
TG: serum triglycerides
TC: total cholesterol
LDL-C: low-density lipoprotein cholesterol
ALT: alanine transferase
AST: aspartic acid transaminase
FBS: fetal bovine serum
OA: oleic acid.
