# Supplementary material for: Liraglutide Alleviates Hepatic Steatosis by Activating the TFEB-Regulated Autophagy-Lysosomal Pathway
Source: Front Cell Dev Biol. 2020 Nov 27;8:602574. doi: 10.3389/fcell.2020.602574 (PMC7729067; doi:10.3389/fcell.2020.602574)
Supplement: Supplementary file 1 [file Data_Sheet_1.PDF]

Supplementary Material

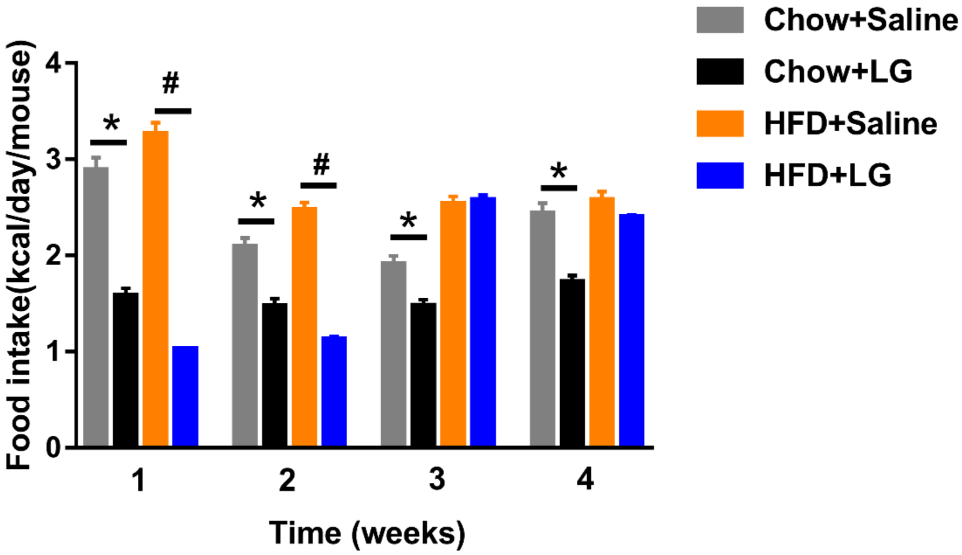

Supplementary Figure 1. Food intake during treatment (kcal/day/mouse). The data are expressed as mean ± S.E.M; n=3-5. $^{*}P<0.01$ vs. Chow+saline group; $^{\#}P<0.01$ vs. HFD+saline group.

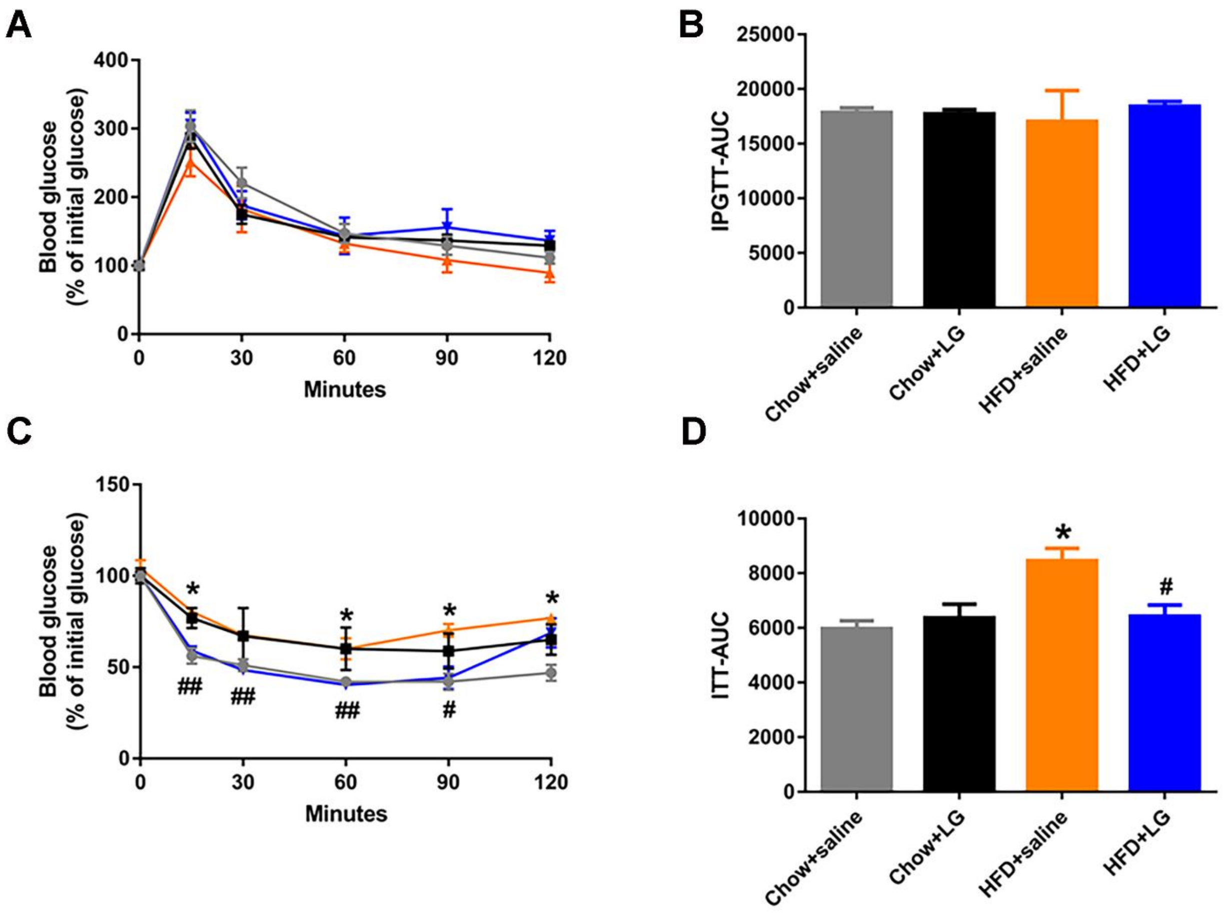

Supplementary Figure 2. (A) IPGTT results were obtained after liraglutide treatment. (B) AUC of IPGTT. AUC: the area under the curve. (C) ITT results were obtained after liraglutide treatment. (D) AUC of ITT. The data are expressed as mean ± S.E.M; n=3-5. $^{*}P$<0.01 vs. Chow+saline group; $^{\#}P$<0.01, $^{\#\#}P$<0.05 vs. HFD+saline group.

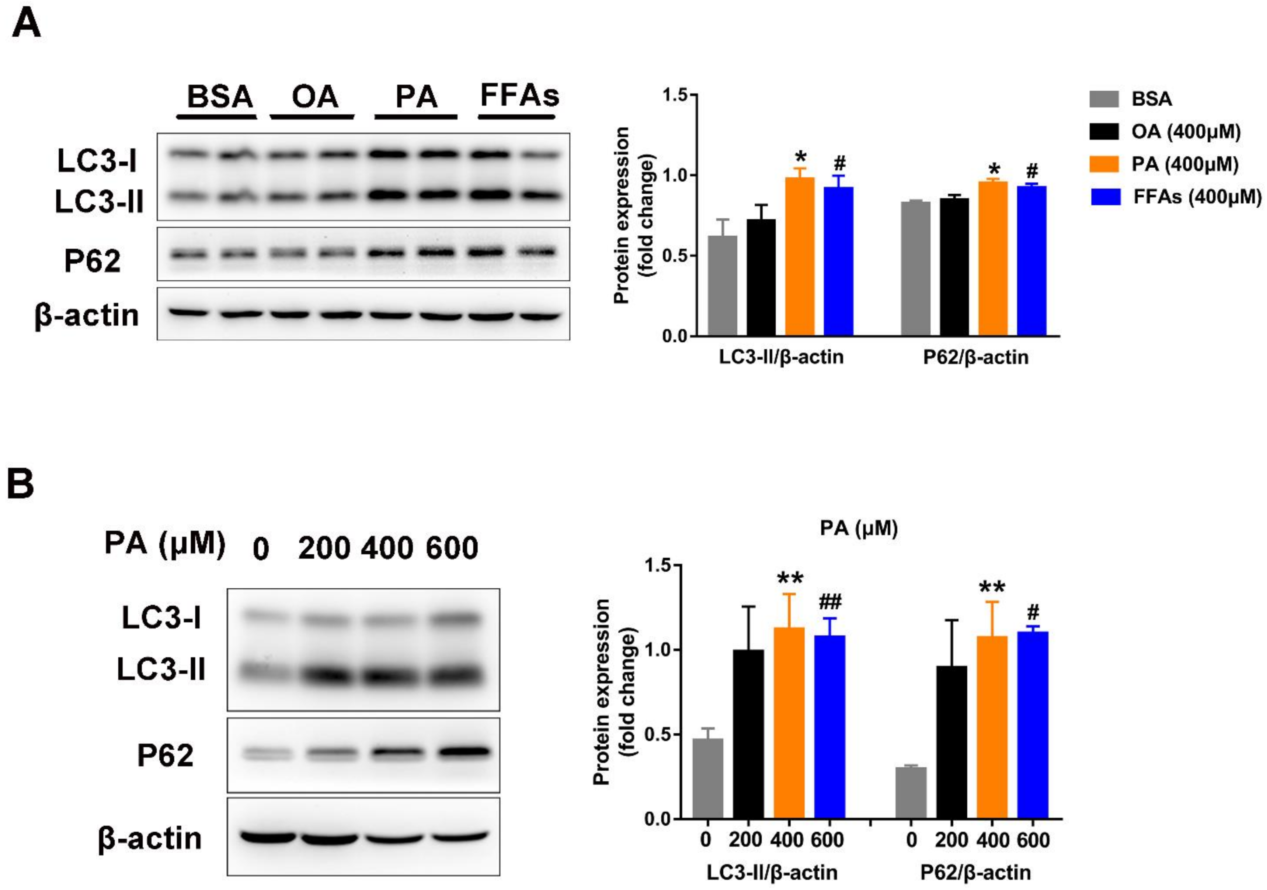

Supplementary Figure 3. (A) Western blot analysis of autophagy-related proteins LC3II and autophagic selective substrate p62 in primary mouse hepatocytes treated with 5 % BSA, OA (400 μM), PA (400 μM) or FFAs (400 μM) for 24 h. (B) Western blot analysis of LC3II and P62 expression in PA-stimulated primary hepatocytes in a dose-dependent manner. The data are expressed as mean ± S.E.M; n=3. $^{*}P<0.01$, $^{**}P<0.05$, $^{\#}P<0.01$ $^{\#\#}P<0.05$ vs. BSA group.

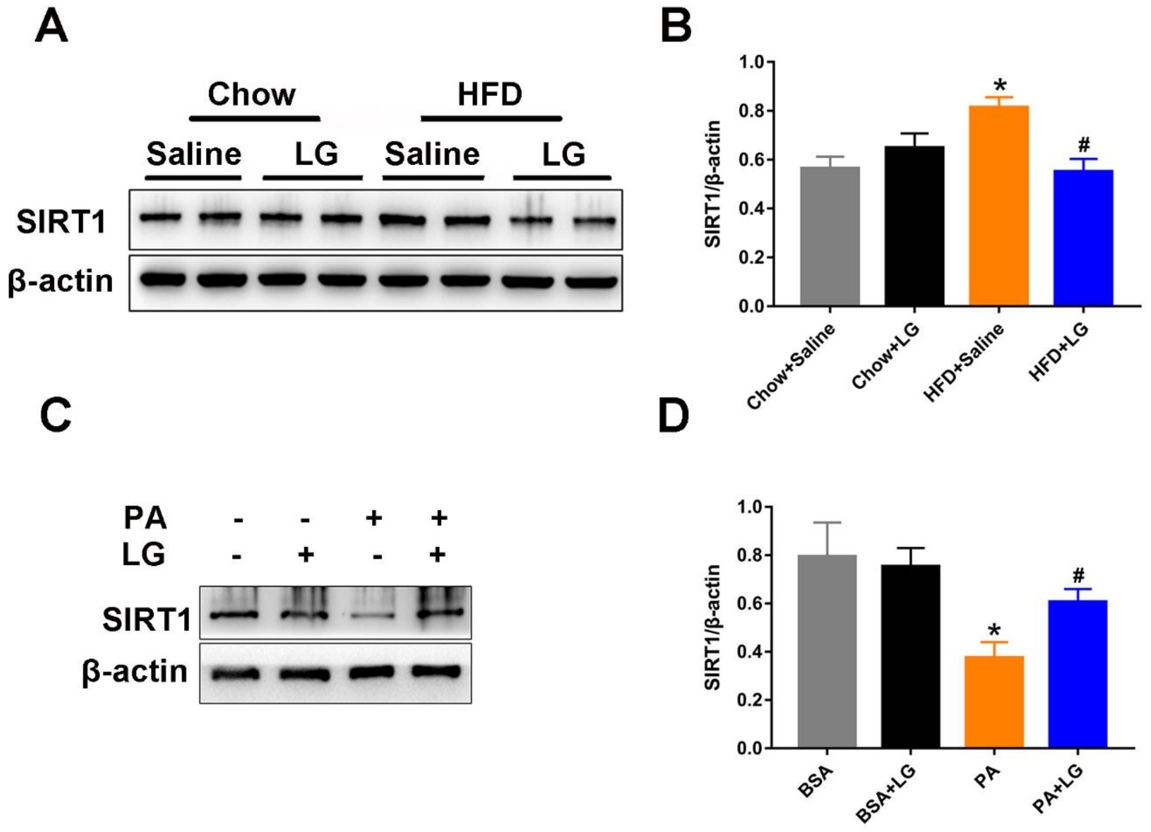

Supplementary Figure 4. (A,B) Western blot analysis of SIRT1 expression in the liver of HFD mice treated with or without liraglutide. Relative expression levels were normalized to β-actin (C,D) Western blot analysis of SIRT1 expression in PA-stimulated primary hepatocytes. The data are expressed as mean ± S.E.M; n=3. $^{*}P<0.01$ vs. Chow+saline and BSA group; $^{\#}P<0.01$ vs. HFD+saline and PA group.
